# The impact of trust in government on pandemic management on the compliance with voluntary COVID-19 vaccination policy among adolescents after social unrest in Hong Kong

**DOI:** 10.3389/fpubh.2022.992895

**Published:** 2022-09-16

**Authors:** Gary Ka-Ki Chung, Yat-Hang Chan, Siu-Ming Chan, Ji-Kang Chen, Hung Wong, Roger Yat-Nork Chung

**Affiliations:** ^1^CUHK Institute of Health Equity, The Chinese University of Hong Kong, Hong Kong, China; ^2^Department of Social and Behavioural Sciences, The City University of Hong Kong, Hong Kong, China; ^3^Department of Social Work, The Chinese University of Hong Kong, Hong Kong, China; ^4^The Jockey Club School of Public Health and Primary Care, The Chinese University of Hong Kong, Hong Kong, China; ^5^CUHK Centre for Bioethics, The Chinese University of Hong Kong, Hong Kong, China

**Keywords:** COVID-19, vaccination, willingness, intention, trust, Hong Kong, adolescents

## Abstract

**Background:**

The launch of COVID-19 vaccines among students provides an opportunity to re-open schools safely. Nonetheless, under the voluntary vaccination policy, the lack of trust in government since the unprecedented massive social unrest in Hong Kong may hinder the vaccination progress. This study aims to assess the impact of trust in government regarding pandemic management on the willingness, uptake, and intention of COVID-19 vaccination among students in Hong Kong.

**Methods:**

Based on maximum variation sampling of 12 secondary schools of diverse socioeconomic background, 1,020 students aged 14–16 years completed an online survey between September and October 2021.

**Results:**

59.2% of the sample received at least one dose of the COVID-19 vaccine, 25.2% showed willingness of vaccination, 44.7% of the unvaccinated intended to receive the vaccine, whereas 13.4% were trustful to the government regarding pandemic management. Results from multivariable logistic regressions showed independent associations of trust with greater vaccination uptake [aOR = 1.63 (95% CI = 1.06–2.52), compared to distrust], willingness [aOR = 12.40 (7.72–19.93)], and intention [aOR = 4.49 (2.06–9.75)]. However, the impact of trust on vaccine uptake reversed [aOR = 0.53 (0.32–0.87)] after additional adjustment for the willingness of vaccination.

**Conclusion:**

Students with higher trust in government regarding pandemic management tended to have greater vaccination willingness and hence uptake; nonetheless, given the same level of willingness, distrust might have facilitated a faster adoption of vaccination as a self-initiated protective behavior. As the level of trust is generally low among secondary school students in Hong Kong, rebuilding trust during adolescence is of importance for better preparedness of the next pandemic.

## Introduction

Prolonged school closure and education disruption has been a major challenge under the COVID-19 pandemic (1). Given the launch and increasing supply of COVID-19 vaccines, most countries strived to boost the COVID-19 vaccination rate among school children, in hope to scale down further class suspensions and re-open schools safely (2). Although vaccination could be executed as a mandatory policy, adopting an opt-in approach could bypass foreseeable ethical and political challenges, particularly in more democratic societies (3). Nonetheless, to achieve a high vaccination rate under such optional policy, the willingness and intention of the public to be vaccinated become crucial (1, 4, 5). Unfortunately, the intention of COVID-19 vaccination was generally lower among young people than the general population (1), possibly for several reasons. First, they usually have a lower risk of severe COVID-19 compared with the older adults (6); second, their decision on vaccination was substantially affected by parents and family who had higher status within the households (2, 7). Previous studies showed that vaccine hesitancy among parents was associated with their scientific knowledge of vaccines as well as their educational levels, which could in turn hinder their children's willingness and intention to receive vaccine (1, 8).

While most existing studies investigated vaccination acceptance and uptake of school children from the perspective of parents as their proxy decision makers, it is at least equally important to understand the predictors of the intention and uptake of COVID-19 vaccines among school children themselves as their preference and decision on vaccination, in particular among adolescents, should be valued (9). This is a gap in knowledge that we aim to address in the present study. Apart from factors such as worries about COVID-19 infection and vaccination, physical fitness, social deprivation, and sense of belonging at school and the community (10, 11), their attitude toward the government was also found as a key factor to the compliance with vaccination and other COVID-19 containment measures imposed by the government in the literature (12). In general, the higher the level of trust, the greater the willingness and intention of vaccination would be (13–17). Nonetheless, previous cross-country research showed that younger people tend to have lower trust in their governments (18). A recent study in Israel also explicitly pointed that distrust in government is one of the major reasons for adolescents not being vaccinated (19). Despite a common phenomenon across the globe, an investigation into the impact of trust on COVID-19 vaccination among adolescents is particularly meaningful due to the sociopolitical context of Hong Kong as it had experienced months of unprecedented social unrest [i.e., the anti-extradition bill social movement in 2019 right before the COVID-19 pandemic (20–22)], in which young people, including secondary school students, were the major participants (23, 24). Hence, their trust in government were inevitably undermined, possibly lowering their willingness to comply with the vaccination policies.

Under the unique sociopolitical context of Hong Kong, the present study aims to examine the impact of trust in government regarding pandemic management and other potential predictors on vaccination willingness and uptake among secondary school students, and to assess their influences on the intention of vaccination among those who have yet been vaccinated.

## Materials and methods

### Study population

Data were collected from a purposive sample of different socioeconomic background in Hong Kong *via* online survey between September and October 2021. The socioeconomic background of interested schools, supplemented with their academic intake, was predicted by the Programme for International Student Assessment (PISA) index of economic, social, and cultural status (ESCS), which was derived from parental education, highest parental occupation, and home possessions. Invitation letters were sent to members of the Hong Kong Association of the Heads of Secondary Schools to recruit all Secondary 3 students (equivalent to Grade 9 in the United States or Year 10 in the United Kingdom) enrolled in each participating school. Eventually, 12 secondary schools of different PISA index of ESCS were selected to participate in the survey.

Among the 1,467 enrolled Secondary 3 students in the 12 participating schools, 1,254 students were successfully surveyed with a response rate of 85.48%. According to the pre-determined inclusion criteria, 1,095 students aged 14–16 years who consented to participate were eligible for this study. After exclusion of 75 cases with missing data, 1,020 students were included for analysis. This study has been approved by the Survey and Behavioral Research Ethics Committee of the Chinese University of Hong Kong in May 2021 (Ref. No. SBRE-20-719).

### Measurements

#### Uptake and intention of COVID-19 vaccination

Participants were asked if they had received at least one dose of COVID-19 vaccine. If not, they were followed up with the question “if the Government would provide a free-of-charge COVID-19 vaccine within the next 12 months, will you receive it?” with four ordinal options (i.e., 1 = definitely yes, 2 = probably yes, 3 = probably not, 4 = definitely not). Their responses were then grouped into having “intention” and “no intention” to receive the vaccine for analysis.

#### Willingness of vaccination

Participants were asked “from your personal perspective, how willing are you to receive COVID-19 vaccination?” with five ordinal options (i.e., 1 = very unwilling, 2 = somewhat unwilling, 3 = neutral, 4 = somewhat willing, 5 = very willing). The responses were then grouped into “unwilling,” “neutral,” and “willing” to receive the vaccine.

#### Trust in government on pandemic management

Participants were asked “how much do you trust the government to take care of its citizens during the COVID-19 pandemic?” with five ordinal options (i.e., 1 = distrust completely, 2 = distrust somewhat, 3 = neither trust nor distrust, 4 = trust somewhat, 5 = trust completely). The responses were then grouped into “distrust,” “neutral,” and “trust.”

#### Demographic characteristics and socioeconomic position

The self-perceived family's socioeconomic position of respondents was measured using the first item of the MacArthur Scale of Subjective Social Status—Youth Version (25). Respondents were asked to mark the rung that best represents where their family would be on a socioeconomic ladder ranging from rung 1 (the worst off) to rung 10 (the best off). Gender, household size, and parental educational levels were regarded as potential confounding factors while age was asked for inclusion selection. Parental educational level was classified as “primary level and below,” “lower secondary level,” “upper secondary level/non-tertiary post-secondary level,” “tertiary level/post-graduate level,” and “N.A.,” which included those who did not know the educational levels of their parents or did not have a male/female guardian. As for family's financial difficulty, respondents were asked to what extent the changes related to the COVID-19 outbreak have created financial problems for their family with five ordinal options (i.e., 1 = not at all; 2 = slightly; 3 = moderately; 4 = very; 5 = extremely).

#### Physical and psychosocial status

For physical fitness, the participants were asked if they had been assessed by a doctor that their health conditions might not be fit for COVID-19 vaccination. For psychosocial status, the participants were assessed on their (i) level of loneliness, (ii) overall worry about COVID-19, (iii) mental health status during the pandemic, (iv) life satisfaction, and (v) resilience. Loneliness was measured using the UCLA 3-item loneliness scale (26) on (i) feeling that the respondents lack companionship, (ii) feeling left out, and (iii) feeling isolated from others, each with three ordinal options (i.e., 1 = hardly ever; 2 = some of the time; 3 = often). The summated score was then re-grouped into “not lonely” (scored 5 or below) and “lonely” (scored 6–9). Regarding overall worry about COVID-19, respondents were asked how worried they were about the local COVID-19 situation with five ordinal options (i.e., 1 = not at all; 2 = slightly; 3 = moderately; 4 = very; 5 = extremely). Mental health status during the pandemic were measured by the revised Mental Health Inventory-5 (MHI-5), which is a brief version that comprises five items from the original 38 items that reproduce a total score ranging from 0 to 15 (27). The MHI-5 score was classified into “severe” (scored 0–8), “mild” (scored 9–10), and “normal” (scored 11–15) (28). About life satisfaction, the participants were asked to rate their life satisfaction during the pandemic from 1 (the worst off) to 10 (the best off). Resilience was measured using the 6-item Brief Resilience Scale (29). The score was grouped into “low resilience” (scored 1.00–2.99), “normal resilience” (scored 3.00–4.30), and “high resilience” (scored 4.31–5.00).

### Statistical analysis

Continuous variables were reported as mean and standard deviation (SD), while categorical variables were reported as count data and percentages. The association between trust in government on pandemic management and willingness to receive the COVID-19 vaccine was first examined using multivariable ordinal logistic regression. Then, multivariable binary logistic regression was performed to examine the predictors of COVID-19 vaccination, with progressive confounding adjustments in two multivariable models. The first multivariable model was to test the statistical significance of trust in government on pandemic management, socioeconomic position, the family's financial difficulty, and demographic characteristics on predicting actual COVID-19 vaccination. The willingness to be vaccinated was then included in the second multivariable model for further examination. Likelihood ratio test was performed to assess the relevance of including willingness to be vaccinated in the regression model. In addition, the same progressive multivariable binary logistic regression analysis was replicated to examine the predictors of the intention of vaccination among the non-vaccinated respondents. The potential multicollinearity effects between predictors of COVID-19 vaccination were examined using generalized variance inflation factor at a conservative threshold of 5 (30). Sensitivity analyses using continuous variables for scores and original categorization for Likert questions with no re-grouping were also performed to ensure the robustness of the results. All data analyses were conducted using SPSS version 26 and RStudio version 1.4.1103. Odds ratios (OR) and 95% confidence interval (CI) are presented, and all statistical tests were two-tailed with a significance level of *p* < 0.05.

## Results

Among 1,020 eligible cases, the majority (82.8%) was aged 14 years and slightly over half (54.0%) were female as shown in Table 1. 13.4% reported trust in government to take care of citizens during the pandemic, whereas 34.0% showed distrust. Regarding COVID-19 vaccination, 4.7% were assessed by a doctor that their health conditions may not be fit for vaccination. While 59.2% received at least one dose of the COVID-19 vaccine, only 25.2% reported that they were willing to be vaccinated. In addition, among those who have yet been vaccinated, merely 44.7% intended to receive the vaccine.

**Table 1:** Descriptive statistics of respondents (*n* = 1,020).

	***N* or Mean**	**% or SD**
**Willingness to receive COVID-19 vaccine**		
Unwilling	267	26.2%
Neutral	496	48.6%
Willing	257	25.2%
**Received at least one dose of COVID-19 vaccine**		
Yes	604	59.2%
No	416	40.8%
**Intention to receive COVID-19 vaccine among those not vaccinated (*****n*** **=** **416)**		
Yes	186	44.7%
No	230	55.3%
**Trust in government on pandemic management**		
Distrust	349	34.0%
Neutral	534	52.1%
Trust	137	13.4%
**Socioeconomic position**	5.41	1.67
**Age**		
14	845	82.8%
15	141	13.8%
16	34	3.3%
**Gender**		
Male	469	46.0%
Female	551	54.0%
**Household size**		
1	15	1.5%
2	60	5.9%
3	228	22.4%
4	394	38.6%
5	216	21.2%
6 or more	107	10.5%
**Father's educational attainment**		
Primary level and below	36	3.5%
Lower secondary level	183	17.9%
Upper secondary level/non-tertiary post-secondary level	289	28.3%
Tertiary level/post-graduate level	194	19.0%
N.A.	318	31.2%
**Mother's educational attainment**		
Primary level and below	66	6.5%
Lower secondary level	170	16.7%
Upper secondary level/non-tertiary post-secondary level	330	32.4%
Tertiary level/post-graduate level	168	16.5%
N.A.	286	28.0%
**Financial difficulty**		
Not at all	261	25.6%
Slightly	352	34.5%
Moderately	312	30.6%
Very	74	7.3%
Extremely	21	2.1%
**Physical fitness for vaccination**		
Fit	972	95.3%
Unfit	48	4.7%
**Loneliness**		
Not lonely	698	68.4%
Lonely	322	31.6%
**Overall worry about the COVID-19 pandemic**		
Not at all worried	184	18.0%
Slightly worried	396	38.8%
Moderately worried	310	30.4%
Very worried	86	8.4%
Extremely worried	44	4.3%
**Mental health status during the pandemic**		
Severe mental health problem	348	34.1%
Mild mental health problem	315	30.9%
Normal mental health	357	35.0%
**Life satisfaction**	5.82	2.33
**Resilience**		
Low	282	27.6%
Normal	677	66.4%
High	61	6.0%

In Table 2, significant independent associations were found for being trustful [aOR = 12.40 (95% CI = 7.72–19.93), *p* < 0.001] and neutral [aOR = 3.37 (95% CI = 2.55–4.45), *p* < 0.001] with greater willingness to be vaccinated, as compared with those having distrust. In Table 3, results from binary logistic regression showed an unadjusted measure of association of trust in government on pandemic management with COVID-19 vaccination [OR = 1.65 (95% CI = 1.09–2.50), *p* = 0.017]. Also, compared with those unwilling to be vaccinated, respondents who were willing [OR = 11.42 (7.47–17.44), *p* < 0.001] and neutral [OR = 3.25 (2.38–4.45), *p* < 0.001] to receive the vaccine were significantly associated with COVID-19 vaccination. After adjustments for confounders, the effect of trust in government on pandemic management remained statistically significant [aOR = 1.63 (1.06–2.52), *p* = 0.026 in Adjusted Model 1]. However, with additional inclusion of the willingness to be vaccinated in Adjusted Model 2, the effect of trust reversed in direction while that of willingness to be vaccinated remained strong and positive. Specifically, participants who were trustful [aOR = 0.53 (0.32–0.87), *p* = 0.013] and neutral [aOR = 0.71 (0.51–0.98), *p* = 0.039] to the government regarding pandemic management were inversely associated with COVID-19 vaccination when taking their willingness into account. Result of likelihood ratio test showed a significant decrease in deviance [−159.88 (Δdf = 2); *p* < 0.001] after including willingness to be vaccinated for additional adjustment, suggesting an improvement of explanatory power in Adjusted Model 2.

**Table 2:** Multivariable ordinal logistic regression on predictors of the willingness to receive the COVID-19 vaccine (*n* = 1,020).

	**Unadjusted model** **OR (95% CI)**	**Adjusted model^a^** **aOR (95% CI)**
**Trust in government on pandemic management**		
Distrust	1	1
Neutral	3.27 (2.49–4.29)***	3.37 (2.55–4.45)***
Trust	13.86 (8.88–21.63)***	12.40 (7.72–19.93)***

* p <0.05; ** p <0.01; *** p <0.001.

^a^ The adjusted model included socioeconomic position, age, gender, household size, father's educational attainment, mother's educational attainment, financial difficulty, physical fitness for vaccination, loneliness, overall worry about the COVID-19 pandemic, mental health status during the pandemic, life satisfaction, and resilience.

**Table 3:** Multivariable logistic regression on predictors of COVID-19 vaccination (*n* = 1,020).

	**Unadjusted model** **OR (95% CI)**	**Adjusted model 1** **aOR (95% CI)**	**Adjusted model 2** **aOR (95% CI)**
**Trust in government on pandemic management**			
Distrust	1	1	1
Neutral	1.20 (0.91–1.58)	1.23 (0.93–1.64)	0.71 (0.51–0.98)*
Trust	1.65 (1.09–2.50)*	1.63 (1.06–2.52)*	0.53 (0.32–0.87)*
**Socioeconomic position**	1.00 (0.92–1.07)	1.01 (0.93–1.09)	0.99 (0.91–1.09)
**Age**			
14	1	1	1
15	1.08 (0.75–1.55)	1.03 (0.71–1.52)	1.11 (0.74–1.67)
16	0.78 (0.39–1.54)	0.64 (0.32–1.32)	0.52 (0.23–1.15)
**Gender**			
Male	1	1	1
Female	0.83 (0.65–1.07)	0.80 (0.61–1.06)	0.95 (0.70–1.27)
**Household size**			
1	1	1	1
2	0.86 (0.26–2.85)	0.76 (0.21–2.82)	0.89 (0.22–3.63)
3	0.66 (0.22–2.00)	0.61 (0.18–2.04)	0.83 (0.23–3.06)
4	0.72 (0.24–2.16)	0.64 (0.19–2.11)	0.80 (0.22–2.87)
5	0.71 (0.24–2.16)	0.62 (0.18–2.07)	0.76 (0.21–2.78)
6 or more	0.80 (0.26–2.52)	0.74 (0.21–2.56)	0.94 (0.25–3.57)
**Father's educational attainment**			
Primary level and below	1	1	1
Lower secondary level	0.74 (0.35–1.56)	0.76 (0.34–1.72)	1.03 (0.39–2.73)
Upper secondary level/non-tertiary post-secondary level	0.88 (0.42–1.82)	1.01 (0.46–2.23)	1.28 (0.49–3.31)
Tertiary level/post-graduate level	0.62 (0.29–1.30)	0.68 (0.29–1.57)	0.72 (0.26–1.95)
N.A.	0.65 (0.32–1.35)	0.71 (0.31–1.62)	0.89 (0.33–2.42)
**Mother's educational attainment**			
Primary level and below	1	1	1
Lower secondary level	0.96 (0.53–1.73)	0.91 (0.49–1.67)	0.84 (0.44–1.59)
Upper secondary level/non-tertiary post-secondary level	0.72 (0.41–1.24)	0.69 (0.38–1.24)	0.65 (0.35–1.21)
Tertiary level/post-graduate level	0.81 (0.45–1.46)	0.85 (0.43–1.67)	0.76 (0.37–1.58)
N.A.	0.70 (0.40–1.22)	0.78 (0.42–1.46)	0.76 (0.39–1.48)
**Financial difficulty**			
Not at all	1	1	1
Slightly	1.12 (0.81–1.55)	1.09 (0.78–1.53)	1.01 (0.70–1.46)
Moderately	1.16 (0.83–1.62)	1.14 (0.80–1.63)	0.95 (0.65–1.41)
Very	1.64 (0.95–2.83)	1.61 (0.90–2.89)	1.40 (0.73–2.66)
Extremely	1.97 (0.74–5.23)	1.98 (0.70–5.55)	1.72 (0.67–4.38)
**Physical fitness for vaccination**			
Fit	1	1	1
Unfit	0.62 (0.35–1.11)	0.62 (0.35–1.11)	0.72 (0.38–1.38)
**Loneliness**			
Not lonely	1	1	1
Lonely	1.03 (0.78–1.34)	1.06 (0.78–1.43)	0.99 (0.91–1.09)
**Overall worry about the COVID-19 pandemic**			
Not at all worried	1	1	1
Slightly worried	1.13 (0.79–1.61)	1.11 (0.76–1.62)	1.04 (0.70–1.57)
Moderately worried	0.98 (0.68–1.42)	0.96 (0.64–1.42)	0.78 (0.51–1.20)
Very worried	1.05 (0.62–1.76)	0.99 (0.56–1.75)	0.99 (0.54–1.81)
Extremely worried	0.95 (0.49–1.84)	0.93 (0.46–1.85)	1.04 (0.48–2.24)
**Mental health status during the pandemic**			
Severe mental health problem	1	1	1
Mild mental health problem	1.07 (0.78–1.46)	1.00 (0.71–1.39)	1.02 (0.71–1.45)
Normal mental health	0.86 (0.64–1.16)	0.76 (0.54–1.08)	0.70 (0.48–1.02)
**Life satisfaction**	1.01 (0.95–1.06)	1.00 (0.94–1.06)	0.98 (0.92–1.04)
**Resilience**			
Low	1	1	1
Normal	1.19 (0.90–1.57)	1.23 (0.90–1.69)	1.04 (0.74–1.47)
High	1.04 (0.60–1.82)	1.19 (0.64–2.20)	1.01 (0.51–2.00)
**Willingness to receive COVID-19 vaccine**			
Unwilling	1		1
Neutral	3.25 (2.38–4.45)***		3.80 (2.68–5.41)***
Willing	11.42 (7.47–17.44)***		17.29 (10.59–28.25)***

* p <0.05; ** p <0.01; *** p <0.001.

The same approach of multivariable binary logistic regression was performed to test the predictors of intention to receive the vaccine among those not vaccinated (Table 4). In Unadjusted Model, being trustful [OR = 4.69 (2.30–9.59), *p* < 0.001] and neutral [OR = 2.02 (1.32–3.11), *p* = 0.001] to the government regarding pandemic management were statistically significant. The effect of father's educational attainment was statistically significant in the groups of lower secondary level [OR = 0.20 (0.05–0.81), *p* = 0.024] and upper secondary level/non-tertiary post-secondary level [OR = 0.25 (0.06–0.98), *p* = 0.046]. The effect of life satisfaction was also positively associated with the vaccination intention [OR = 1.11 (1.02–1.20), *p* = 0.021]. In Adjusted Model 1, being trustful [aOR = 4.49 (2.06–9.75), *p* < 0.001] and neutral [aOR = 1.97 (1.23–3.16), *p* = 0.005] to the government regarding pandemic management remained statistically significant, as did the effect of life satisfaction [aOR = 1.12 (1.00–1.24), *p* = 0.045]. Yet, the effect of father's educational attainment was mitigated, and a significant association was found in the group of having moderate financial difficulty [aOR = 1.85 (1.01–3.39), *p* = 0.048]. After the additional adjustment for the willingness to be vaccinated in Adjusted Model 2, no significant association of trust in government regarding pandemic management with the vaccination intention was observed, while those who were willing [aOR = 60.16 (16.53–219.20), *p* < 0.001] and neutral [aOR = 9.93 (5.53–17.85), *p* < 0.001] to receive the vaccine were significantly associated with the vaccination intention. A significantly greater intention was also found in those who were “very worried” about the COVID-19 pandemic [aOR = 3.26 (1.16–9.15), *p* = 0.024]. Again, the observed significant decrease in deviance [−102.08 (Δdf = 2); *p* < 0.001] in Adjusted Model 2 compared to Adjusted Model 1 indicated an improvement of explanatory power of the regression model.

**Table 4:** Multivariable logistic regression on predictors of the intention to receive COVID-19 vaccine among non-vaccinated respondents (*n* = 416).

	**Unadjusted model** **OR (95% CI)**	**Adjusted model 1** **aOR (95% CI)**	**Adjusted model 2** **aOR (95% CI)**
**Trust in government on pandemic management**			
Distrust	1	1	1
Neutral	2.02 (1.32–3.11)**	1.97 (1.23–3.16)**	0.99 (0.55–1.76)
Trust	4.69 (2.30–9.59)***	4.49 (2.06–9.75)***	1.57 (0.62–3.95)
**Socioeconomic position**	0.98 (0.87–1.11)	0.94 (0.80–1.10)	0.88 (0.74–1.05)
**Age**			
14	1	1	1
15	1.27 (0.72–2.24)	1.20 (0.63–2.28)	1.35 (0.61–2.96)
16	2.19 (0.78–6.17)	2.32 (0.73–7.35)	1.98 (0.62–6.26)
**Gender**			
Male	1	1	1
Female	0.90 (0.61–1.34)	1.06 (0.67–1.67)	1.26 (0.76–2.12)
**Household size**			
1	1	1	1
2	0.86 (0.12–6.27)	1.34 (0.22–8.21)	1.26 (0.10–16.56)
3	1.03 (0.17–6.48)	1.58 (0.30–8.23)	2.32 (0.20–27.41)
4	1.34 (0.22–8.24)	2.14 (0.43–10.71)	3.03 (0.27–33.58)
5	1.34 (0.21–8.41)	2.23 (0.43–11.58)	2.60 (0.23–29.70)
6 or more	1.17 (0.18–7.79)	2.02 (0.37–11.17)	2.63 (0.22–31.00)
**Father's educational attainment**			
Primary level and below	1	1	1
Lower secondary level	0.20 (0.05–0.81)*	0.43 (0.10–1.83)	0.84 (0.08–8.66)
Upper secondary level/non-tertiary post-secondary level	0.25 (0.06–0.98)*	0.60 (0.14–2.65)	1.23 (0.13–11.89)
Tertiary level/post-graduate level	0.30 (0.08–1.17)	0.70 (0.15–3.26)	1.53 (0.15–16.15)
N.A.	0.28 (0.07–1.08)	0.71 (0.16–3.23)	1.26 (0.12–12.99)
**Mother's educational attainment**			
Primary level and below	1	1	1
Lower secondary level	1.61 (0.61–4.27)	1.72 (0.66–4.45)	1.21 (0.32–4.57)
Upper secondary level/non-tertiary post-secondary level	0.97 (0.39–2.38)	1.02 (0.41–2.51)	0.65 (0.18–2.36)
Tertiary level/post-graduate level	1.81 (0.69–4.74)	1.80 (0.63–5.16)	1.04 (0.25–4.42)
N.A.	1.28 (0.52–3.18)	1.28 (0.49–3.32)	0.94 (0.24–3.71)
**Financial difficulty**			
Not at all	1	1	1
Slightly	1.44 (0.87–2.37)	1.59 (0.91–2.79)	1.79 (0.95–3.37)
Moderately	1.67 (1.00–2.80)	1.85 (1.01–3.39)*	1.30 (0.67–2.55)
Very	1.42 (0.58–3.44)	2.02 (0.79–5.21)	1.58 (0.55–4.54)
Extremely	0.84 (0.15–4.76)	1.05 (0.15–7.29)	1.85 (0.12–27.49)
**Physical fitness for vaccination**			
Fit	1	1	1
Unfit	0.46 (0.19–1.13)	0.43 (0.15–1.17)	0.47 (0.16–1.43)
**Loneliness**			
Not lonely	1	1	1
Lonely	0.83 (0.54–1.26)	1.05 (0.63–1.76)	0.90 (0.50–1.62)
**Overall worry about the COVID-19 pandemic**			
Not at all worried	1	1	1
Slightly worried	1.05 (0.61–1.84)	0.99 (0.53–1.84)	0.96 (0.44–2.09)
Moderately worried	1.19 (0.67–2.10)	1.44 (0.76–2.71)	1.03 (0.48–2.22)
Very worried	1.88 (0.84–4.21)	2.11 (0.86–5.18)	3.26 (1.16–9.15)*
Extremely worried	1.02 (0.37–2.83)	1.30 (0.40–4.24)	1.79 (0.54–5.92)
**Mental health status during the pandemic**			
Severe mental health problem	1	1	1
Mild mental health problem	0.90 (0.55–1.47)	0.84 (0.49–1.45)	0.68 (0.36–1.27)
Normal mental health	1.18 (0.75–1.87)	0.82 (0.45–1.48)	0.69 (0.35–1.37)
**Life satisfaction**	1.11 (1.02–1.20) *	1.12 (1.00–1.24) *	1.10 (0.98–1.24)
**Resilience**			
Low	1	1	1
Normal	1.50 (0.97–2.32)	1.55 (0.92–2.62)	1.37 (0.73–2.58)
High	1.95 (0.83–4.58)	1.85 (0.63–5.45)	2.59 (0.67–10.05)
**Willingness to receive COVID-19 vaccine**			
Unwilling	1		1
Neutral	8.05 (4.95–13.11)***		9.93 (5.53–17.85)***
Willing	45.29 (15.01–136.73)***		60.16 (16.53–219.20)***

* p <0.05; ** p <0.01; *** p <0.001.

In all the above regression models, multicollinearity effects were small as none of the predictors had a generalized variance inflation factor greater than the threshold of 5. The pattern of results remained consistent in the sensitivity analyses with predictors treated as continuous variables for scores and raw categories for Likert questions (Supplementary Tables 1–3).

## Discussion

To the best of our knowledge, this is the first study to investigate the predictors of the willingness, uptake, and intention of COVID-19 vaccination among secondary school students in Hong Kong. It is worth noting that the present study reifies the significance of trust in government regarding pandemic management among students from the perspective of public health. While previous local research recommended extra attention to tertiary-educated parents who tend to be more critical and distrustful toward the government, and hence resist vaccination recommendations to their children in primary school (2), the effect of parental education level on COVID-19 vaccination was not significant in our sample of secondary school students. In addition to reflecting actual non-association of parental education in vaccination behavior in Hong Kong, the discrepancy could possibly be attributable to the diminishing influence of parents as children grow up. Also, family's socioeconomic position and household size did not predict vaccination behavior among our sampled students, which counters the findings of another previous study where parental vaccine hesitancy was found to be associated with socioeconomic disadvantages (31).

The major and perhaps most interesting finding of this study lies in the conflicting influence of trust in government regarding pandemic management on COVID-19 vaccine uptake before and after the statistical adjustment for willingness of vaccination. The effect of trust on vaccination could manifest at least in two ways under the sociopolitical context in Hong Kong. On one hand, distrust in government was strongly associated with a lower willingness of COVID-19 vaccination and thus a lower vaccine uptake, plausibly due to the widespread discontent among young people as a result of the anti-extradition bill social unrest in 2019 and the resultant unreceptive attitude toward the government and its measures. On the other hand, people who distrust the government also tend to be more proactive in self-initiated protective behaviors and community mobilization under the pandemic, as reported by local studies supporting that the higher the level of distrust in government in a district, the faster the anti-pandemic response was (23, 32). Hence, the conflicting influence of trust on vaccine uptake before and after the adjustment for willingness of vaccination could be because those who distrust the government were also more likely to have taken faster health protection response and to receive vaccination than those who trusted the government given the same level of willingness of vaccination, even though distrust in government apparently lowered the willingness of vaccination in the first place.

Afterall, the positive overall association between trust in government and COVID-19 vaccination suggested that it is important to consider sociopolitical factors when implementing public health policies. Given that the effect of trust remained significant among young people who had yet been vaccinated, it reinforces the notion that people who trust the government would still have a greater intention or willingness to be vaccinated. In places such as Israel (33) and Singapore (34) where vaccination coverage ranks the top across the globe, a high level of trust in government from its people is generally observed—over 50% of Israeli trusted their national government (35), while over 90% of Singaporean agreed that information from official government sources was trustworthy (34). However, our present study found half of our sampled adolescents being neutral (52.1%) and a low proportion trusting the government regarding pandemic management (13.6%) in Hong Kong. It is likely that the distrust in government among adolescents had been progressively built up over recent years. Back in 2017, a local survey of junior secondary school students already revealed a comparatively low level of trust in conventional social institutions including the local and national government (36). Nonetheless, the recent anti-extradition bill social unrest appears to be a critical political crisis that further intensified the widespread public distrust, which might have undermined the responses to COVID-19 and compliance with containment measures (37). Examples that the sentiment of the social unrest was carried over to the pandemic responses in Hong Kong include government's refusal to recommend mask wearing at the early phase of the pandemic under the anti-mask law for deterring masked violent protestors amid the social unrest (37), the banning of mandarin-speaking Chinese customers from dining initiated by some restaurants (38), as well as the strike actions to urge for border closure against mainland Chinese travelers and the supply of personal protection equipment by frontline healthcare workers led by the pro-democratic camp (37). More specifically, a recent local study clearly showed that Hong Kong adults with a opposition political stance and distrust in government had significantly lower support for COVID-19 vaccination program (39), and we speculate that such associations would also apply to adolescents in Hong Kong. Although the same study provided some evidence on the efficacy of positive health expert communication to improve support for vaccination among those with a opposition stance (39), it is unfortunate that ineffective risk communication by the Hong Kong government and inconsistency in COVID-19 advices among health experts were not uncommon during the pandemic (23). Therefore, it seems reasonable to observe the overall low level of trust in government regarding pandemic management and willingness of vaccination among adolescents, especially under such sociopolitical context of Hong Kong.

To improve vaccine uptake and willingness among adolescents under the voluntary vaccination policy, both short-term and long-term strategies are warranted. Given the above-mentioned positive impact of health expert communication on support for vaccination program among the low-trust individuals, policymakers should better utilize health experts in risk communication to boost vaccine uptake among adolescents in the short run. Hoffman et al. (40) suggested that health experts could leverage on the “ABCs” of vaccine communication to promote COVID-19 vaccination among adolescents by (i) actively engaging in social media for real-time surveillance and clarification for misinformation, (ii) building trustworthiness around COVID-19 vaccines by non-judgmental exchange during clinician-patient communication and demonstrating empathy and active listening when validating vaccine-related concerns, and (iii) capitalizing on strengths of adolescents in digital literacy by encouraging critical appraisal of online health information and empowering them on how to interpret and discuss the information with their peers. In the long run, if Hong Kong society continues to be strongly polarized in terms of politics, it would be difficult to garner support and compliance with emergency measures to overcome another unforeseen collective catastrophe (41) since a low level of political trust inevitably comes with damage in the legitimacy of health officials and the credibility of public health policies regardless of their intended goals and benefits (42). Given that adolescence is a critical period to develop political beliefs and hence the attitude toward government policies including public health measures (43), rebuilding the mutual trust between adolescents and the government is beneficial not only for compliance with COVID-19 vaccination but also for other government public policies in general. To this end, the five main policy dimensions (including responsiveness, reliability, integrity, openness, and fairness) proposed by the OECD Trust Framework (44) could be considered as guiding principles for decision-making to enhance trust and solidarity, and strengthen legitimacy and acceptability of pandemic-related measures and other public health policies in the future.

### Limitations

This study has several limitations., First, its cross-sectional design could not capture the effect of trust in the government throughout the entire course of the pandemic on vaccination and focused only on a particular time, especially when the pandemic is still not over yet. No inferences around causality can be drawn as temporality of association cannot be established. Second, the study population was not randomly sampled, limiting its representativeness. Nevertheless, we purposively selected schools that represented a wide range of background in the socioeconomic spectrum. Third, in many previous studies about vaccine acceptance, vaccine knowledge was usually one of the studied variables but was not measured in the present study. However, the age range and educational level of our sampled students was narrow, making their means and channels of information access relatively comparable. Fourth, the choices of vaccine may be a latent variable to further inform the mechanism between trust in government on pandemic management and vaccine acceptance, which was examined in some existing vaccine acceptance studies (13, 45). Particularly in Hong Kong where the aftermath of the social unrest is carried onto the pandemic scenario, the choices of vaccine among those vaccinated would possibly further provide information on the divergent level of trust among people. However, this study does not cover the data of vaccine brand selections. Lastly, conspiracy belief has been identified as an important barrier to COVID-19 vaccination among the adult population (46–48); nonetheless, we could not test its impact among adolescents due to data unavailability. In light of the above limitations, we recommend further studies to consider the influence of conspiracy belief and vaccine brands on compliance with vaccination policy among adolescents. Studies are also warranted to assess whether the complicated relationship between trust in government and COVID-19 vaccination solely happens in Hong Kong as there are other countries in the world that had undergone social movements around the time of the COVID-19 pandemic.

## Conclusion

As a missing piece to understand the uptake and intention of COVID-19 vaccination among secondary school students in Hong Kong, this study provides empirical evidence on the independent and potentially conflicting effect of trust in government on pandemic management, as well as the lack of significant influence of parents and family background, on the uptake and intention of vaccination. Despite the overall positive effect of trust on vaccination, those who distrust the government regarding pandemic management had a faster adoption of COVID-19 vaccines given the same level of willingness of vaccination. Nonetheless, as the level of trust is generally low among secondary school students in Hong Kong, rebuilding their trust in government during adolescence is of paramount importance for better preparedness of and greater resilience against the next pathogen of pandemic potential.

## Data availability statement

The raw data supporting the conclusions of this article will be made available by the authors, without undue reservation.

## Ethics statement

The studies involving human participants were reviewed and approved by the Survey and Behavioral Research Ethics Committee of the Chinese University of Hong Kong. Written informed consent to participate in this study was provided by the participants' legal guardian/next of kin.

## Author contributions

GC and Y-HC contributed to literature search, study design, data analysis, result interpretation, and the write-up of the manuscript. S-MC were responsible for study design, data curation, and result interpretation. J-KC was responsible for study design and provided substantial statistical advice on the analyses. HW and RC oversaw the project as the co-principal investigators, and contributed to study design and result interpretation. All authors critically appraised and approved the manuscript.

## Funding

The authors acknowledge funding support from the Worldwide Universities Network (WUN) Research Development Fund 2020.

## Conflict of interest

The authors declare that the research was conducted in the absence of any commercial or financial relationships that could be construed as a potential conflict of interest.

## Publisher's note

All claims expressed in this article are solely those of the authors and do not necessarily represent those of their affiliated organizations, or those of the publisher, the editors and the reviewers. Any product that may be evaluated in this article, or claim that may be made by its manufacturer, is not guaranteed or endorsed by the publisher.
