# Supplementary material for: The impact of trust in government on pandemic management on the compliance with voluntary COVID-19 vaccination policy among adolescents after social unrest in Hong Kong
Source: Front Public Health. 2022 Sep 16;10:992895. doi: 10.3389/fpubh.2022.992895 (PMC9843607; doi:10.3389/fpubh.2022.992895)
Supplement: Supplementary file 1 [file Data_Sheet_1.docx]

| **Supplementary Table 1. Multivariable ordinal logistic regression on predictors of the willingness to receive the COVID-19 vaccine (n=1,020)** | | | | | |
| --- | --- | --- | --- | --- | --- |
|  |  |  | Unadjusted model  OR (95% CI) |  | Adjusted model 1 ^a^  aOR (95% CI) |
| **Trust in government on pandemic management** | | |  |  |  |
|  |  | Distrust completely | 1 |  | 1 |
|  |  | Distrust somewhat | 2.85 ( 1.88 - 4.33 ) *** |  | 3.09 ( 2.03 - 4.73 ) *** |
|  |  | Neither trust nor distrust | 6.03 ( 4.18 - 8.69 ) *** |  | 6.67 ( 4.59 - 9.71 ) *** |
|  |  | Trust somewhat | 21.39 ( 12.40 - 36.89 ) *** |  | 19.41 ( 11.08 - 34.02 ) *** |
|  |  | Trust completely | 73.70 ( 32.01 - 169.69 ) *** |  | 74.51 ( 29.08 - 190.95 ) *** |

* *p* < 0.05; ** *p* < 0.01; *** *p* < 0.001

^a^ The adjusted model included socioeconomic position, age, gender, household size, father's educational attainment, mother's educational attainment, financial difficulty, physical fitness for vaccination, loneliness, overall worry about the COVID-19 pandemic, mental health status during the pandemic, life satisfaction, and resilience.

| **Supplementary Table 2. Multivariable logistic regression on predictors of COVID-19 vaccination (n=1,020)** | | | | | | | |
| --- | --- | --- | --- | --- | --- | --- | --- |
|  |  |  | Unadjusted model OR (95% CI) |  | Adjusted model 1 OR (95% CI) |  | Adjusted model 2 OR (95% CI) |
| **Trust in government on pandemic management** | | |  |  |  |  |  |
|  |  | Distrust completely | 1 |  | 1 |  | 1 |
|  |  | Distrust somewhat | 1.10 ( 0.72 - 1.68 ) |  | 1.11 ( 0.72 - 1.71 ) |  | 0.57 ( 0.34 - 0.95 ) * |
|  |  | Neither trust nor distrust | 1.26 ( 0.89 - 1.78 ) |  | 1.30 ( 0.91 - 1.86 ) |  | 0.46 ( 0.29 - 0.72 ) ** |
|  |  | Trust somewhat | 1.60 ( 0.96 - 2.67 ) |  | 1.55 ( 0.91 - 2.64 ) |  | 0.32 ( 0.16 - 0.62 ) ** |
|  |  | Trust completely | 2.16 ( 1.01 - 4.62 ) * |  | 2.20 ( 1.01 - 4.77 ) * |  | 0.22 ( 0.09 - 0.59 ) ** |
| **Socioeconomic position** | | | 1.00 ( 0.92 - 1.07 ) |  | 1.00 ( 0.93 - 1.09 ) |  | 0.99 ( 0.90 - 1.09 ) |
| **Age** | | |  |  |  |  |  |
|  |  | 14 | 1 |  | 1 |  | 1 |
|  |  | 15 | 1.08 ( 0.75 - 1.55 ) |  | 1.03 ( 0.70 - 1.51 ) |  | 1.06 ( 0.71 - 1.58 ) |
|  |  | 16 | 0.78 ( 0.39 - 1.54 ) |  | 0.63 ( 0.30 - 1.29 ) |  | 0.46 ( 0.20 - 1.06 ) |
| **Gender** | | |  |  |  |  |  |
|  |  | Male | 1 |  | 1 |  | 1 |
|  |  | Female | 0.83 ( 0.65 - 1.07 ) |  | 0.80 ( 0.60 - 1.06 ) |  | 0.91 ( 0.67 - 1.24 ) |
| **Household size** | | | 0.99 ( 0.90 - 1.09 ) |  | 0.99 ( 0.89 - 1.09 ) |  | 0.97 ( 0.87 - 1.08 ) |
| **Father's educational attainment** | | |  |  |  |  |  |
|  |  | Primary level and below | 1 |  | 1 |  | 1 |
|  |  | Lower secondary level | 0.74 ( 0.35 - 1.56 ) |  | 0.82 ( 0.37 - 1.85 ) |  | 1.12 ( 0.44 - 2.86 ) |
|  |  | Upper secondary level / non-tertiary post-secondary level | 0.88 ( 0.42 - 1.82 ) |  | 1.07 ( 0.48 - 2.35 ) |  | 1.37 ( 0.54 - 3.43 ) |
|  |  | Tertiary level / post-graduate level | 0.62 ( 0.29 - 1.30 ) |  | 0.69 ( 0.30 - 1.60 ) |  | 0.71 ( 0.27 - 1.91 ) |
|  |  | N.A. | 0.65 ( 0.32 - 1.35 ) |  | 0.76 ( 0.33 - 1.72 ) |  | 0.86 ( 0.32 - 2.28 ) |
| **Mother's educational attainment** | | |  |  |  |  |  |
|  |  | Primary level and below | 1 |  | 1 |  | 1 |
|  |  | Lower secondary level | 0.96 ( 0.53 - 1.73 ) |  | 0.93 ( 0.50 - 1.71 ) |  | 0.72 ( 0.37 - 1.38 ) |
|  |  | Upper secondary level / non-tertiary post-secondary level | 0.72 ( 0.41 - 1.24 ) |  | 0.70 ( 0.39 - 1.27 ) |  | 0.56 ( 0.30 - 1.07 ) |
|  |  | Tertiary level / post-graduate level | 0.81 ( 0.45 - 1.46 ) |  | 0.86 ( 0.43 - 1.70 ) |  | 0.64 ( 0.31 - 1.35 ) |
|  |  | N.A. | 0.70 ( 0.40 - 1.22 ) |  | 0.80 ( 0.43 - 1.50 ) |  | 0.74 ( 0.37 - 1.48 ) |
| **Financial difficulty** | | |  |  |  |  |  |
|  |  | Not at all | 1 |  | 1 |  | 1 |
|  |  | Slightly | 1.12 ( 0.81 - 1.55 ) |  | 1.10 ( 0.79 - 1.54 ) |  | 1.00 ( 0.69 - 1.44 ) |
|  |  | Moderately | 1.16 ( 0.83 - 1.62 ) |  | 1.17 ( 0.82 - 1.67 ) |  | 0.93 ( 0.63 - 1.39 ) |
|  |  | Very | 1.64 ( 0.95 - 2.83 ) |  | 1.69 ( 0.95 - 2.99 ) |  | 1.29 ( 0.66 - 2.53 ) |
|  |  | Extremely | 1.97 ( 0.74 - 5.23 ) |  | 2.02 ( 0.74 - 5.57 ) |  | 1.60 ( 0.58 - 4.43 ) |
| **Physical fitness for vaccination** | | |  |  |  |  |  |
|  |  | Fit | 1 |  | 1 |  | 1 |
|  |  | Unfit | 0.62 ( 0.35 - 1.11 ) |  | 0.63 ( 0.35 - 1.12 ) |  | 0.75 ( 0.38 - 1.50 ) |
| **Loneliness** | | | 1.01 ( 0.94 - 1.08 ) |  | 1.02 ( 0.94 - 1.11 ) |  | 0.97 ( 0.89 - 1.06 ) |
| **Overall worry about the COVID-19 pandemic** | | |  |  |  |  |  |
|  |  | Not at all worried | 1 |  | 1 |  | 1 |
|  |  | Slightly worried | 1.13 ( 0.79 - 1.61 ) |  | 1.12 ( 0.77 - 1.64 ) |  | 1.03 ( 0.68 - 1.55 ) |
|  |  | Moderately worried | 0.98 ( 0.68 - 1.42 ) |  | 0.97 ( 0.65 - 1.44 ) |  | 0.80 ( 0.52 - 1.24 ) |
|  |  | Very worried | 1.05 ( 0.62 - 1.76 ) |  | 0.97 ( 0.55 - 1.71 ) |  | 0.91 ( 0.48 - 1.70 ) |
|  |  | Extremely worried | 0.95 ( 0.49 - 1.84 ) |  | 0.92 ( 0.46 - 1.82 ) |  | 0.87 ( 0.39 - 1.97 ) |
| **Mental health status during the pandemic** | | | 0.99 ( 0.94 - 1.04 ) |  | 0.97 ( 0.91 - 1.03 ) |  | 0.94 ( 0.87 - 1.00 ) |
| **Life satisfaction** | | | 1.01 ( 0.95 - 1.06 ) |  | 0.99 ( 0.93 - 1.05 ) |  | 0.97 ( 0.90 - 1.03 ) |
| **Resilience** | | | 1.12 ( 0.93 - 1.34 ) |  | 1.20 ( 0.96 - 1.51 ) |  | 1.07 ( 0.82 - 1.38 ) |
| **Willingness to receive COVID-19 vaccine** | | |  |  |  |  |  |
|  |  | Very unwilling | 1 |  |  |  | 1 |
|  |  | Somewhat unwilling | 3.27 ( 1.85 - 5.80 ) *** |  |  |  | 4.36 ( 2.34 - 8.13 ) *** |
|  |  | Neutral | 6.83 ( 4.12 - 11.35 ) *** |  |  |  | 10.97 ( 6.10 - 19.73 ) *** |
|  |  | Somewhat willing | 17.20 ( 9.43 - 31.41 ) *** |  |  |  | 34.23 ( 17.05 - 68.72 ) *** |
|  |  | Very willing | 71.74 ( 25.87 - 198.94 ) *** |  |  |  | 192.29 ( 57.00 - 648.07 ) *** |

* *p* < 0.05; ** *p* < 0.01; *** *p* < 0.001

| **Supplementary Table 3. Multivariable logistic regression on predictors of the intention to receive COVID-19 vaccine among non-vaccinated respondents (n=416)** | | | | | | | |
| --- | --- | --- | --- | --- | --- | --- | --- |
|  |  |  | Unadjusted OR (95% CI) |  | Adjusted Model 1 OR (95% CI) |  | Adjusted Model 2 OR (95% CI) |
| **Trust in government on pandemic management** | | |  |  |  |  |  |
|  |  | Distrust completely | 1 |  | 1 |  | 1 |
|  |  | Distrust somewhat | 1.48 ( 0.75 - 2.91 ) |  | 1.35 ( 0.65 - 2.78 ) |  | 0.41 ( 0.17 - 1.03 ) |
|  |  | Neither trust nor distrust | 2.47 ( 1.41 - 4.33 ) ** |  | 2.30 ( 1.26 - 4.22 ) ** |  | 0.50 ( 0.24 - 1.05 ) |
|  |  | Trust somewhat | 4.75 ( 2.01 - 11.20 ) *** |  | 4.24 ( 1.67 - 10.80 ) ** |  | 0.67 ( 0.23 - 1.97 ) |
|  |  | Trust completely | 11.66 ( 2.33 - 58.26 ) ** |  | 12.19 ( 2.77 - 53.79 ) ** |  | 1.84 ( 0.19 - 17.81 ) |
| **Socioeconomic position** | | | 0.98 ( 0.87 - 1.11 ) |  | 0.94 ( 0.80 - 1.10 ) |  | 1.12 ( 0.99 - 1.26 ) |
| **Age** | | |  |  |  |  |  |
|  |  | 14 | 1 |  | 1 |  | 1 |
|  |  | 15 | 1.27 ( 0.72 - 2.24 ) |  | 1.19 ( 0.62 - 2.28 ) |  | 1.26 ( 0.58 - 2.72 ) |
|  |  | 16 | 2.19 ( 0.78 - 6.17 ) |  | 2.33 ( 0.74 - 7.36 ) |  | 1.45 ( 0.41 - 5.18 ) |
| **Gender** | | |  |  |  |  |  |
|  |  | Male | 1 |  | 1 |  | 1 |
|  |  | Female | 0.90 ( 0.61 - 1.34 ) |  | 1.08 ( 0.68 - 1.69 ) |  | 1.23 ( 0.74 - 2.03 ) |
| **Household size** | | | 1.04 ( 0.90 - 1.20 ) |  | 1.11 ( 0.95 - 1.30 ) |  | 1.11 ( 0.92 - 1.33 ) |
| **Father's educational attainment** | | |  |  |  |  |  |
|  |  | Primary level and below | 1 |  | 1 |  | 1 |
|  |  | Lower secondary level | 0.20 ( 0.05 - 0.81 ) * |  | 0.40 ( 0.09 - 1.79 ) |  | 1.24 ( 0.14 - 10.94 ) |
|  |  | Upper secondary level / non-tertiary post-secondary level | 0.25 ( 0.06 - 0.98 ) * |  | 0.56 ( 0.12 - 2.51 ) |  | 2.00 ( 0.24 - 16.81 ) |
|  |  | Tertiary level / post-graduate level | 0.30 ( 0.08 - 1.17 ) |  | 0.64 ( 0.14 - 3.04 ) |  | 2.11 ( 0.23 - 19.24 ) |
|  |  | N.A. | 0.28 ( 0.07 - 1.08 ) |  | 0.65 ( 0.14 - 2.99 ) |  | 1.65 ( 0.19 - 14.44 ) |
| **Mother's educational attainment** | | |  |  |  |  |  |
|  |  | Primary level and below | 1 |  | 1 |  | 1 |
|  |  | Lower secondary level | 1.61 ( 0.61 - 4.27 ) |  | 1.97 ( 0.71 - 5.44 ) |  | 1.11 ( 0.24 - 5.16 ) |
|  |  | Upper secondary level / non-tertiary post-secondary level | 0.97 ( 0.39 - 2.38 ) |  | 1.23 ( 0.47 - 3.19 ) |  | 0.53 ( 0.12 - 2.32 ) |
|  |  | Tertiary level / post-graduate level | 1.81 ( 0.69 - 4.74 ) |  | 1.95 ( 0.65 - 5.82 ) |  | 0.83 ( 0.16 - 4.35 ) |
|  |  | N.A. | 1.28 ( 0.52 - 3.18 ) |  | 1.55 ( 0.57 - 4.19 ) |  | 0.90 ( 0.20 - 4.05 ) |
| **Financial difficulty** | | |  |  |  |  |  |
|  |  | Not at all | 1 |  | 1 |  | 1 |
|  |  | Slightly | 1.44 ( 0.87 - 2.37 ) |  | 1.55 ( 0.88 - 2.73 ) |  | 1.69 ( 0.88 - 3.24 ) |
|  |  | Moderately | 1.67 ( 1.00 - 2.80 ) |  | 1.91 ( 1.05 - 3.50 ) * |  | 1.38 ( 0.67 - 2.83 ) |
|  |  | Very | 1.42 ( 0.58 - 3.44 ) |  | 1.99 ( 0.77 - 5.09 ) |  | 1.37 ( 0.48 - 3.92 ) |
|  |  | Extremely | 0.84 ( 0.15 - 4.76 ) |  | 1.17 ( 0.16 - 8.45 ) |  | 1.60 ( 0.09 - 28.96 ) |
| **Physical fitness for vaccination** | | |  |  |  |  |  |
|  |  | Fit | 1 |  | 1 |  | 1 |
|  |  | Unfit | 0.46 ( 0.19 – 1.12 ) |  | 0.43 ( 0.16 - 1.18 ) |  | 0.54 ( 0.17 – 1.75 ) |
| **Loneliness** | | | 0.97 ( 0.88 - 1.08 ) |  | 1.06 ( 0.92 - 1.21 ) |  | 1.03 ( 0.88 - 1.20 ) |
| **Overall worry about the COVID-19 pandemic** | | |  |  |  |  |  |
|  |  | Not at all worried | 1 |  | 1 |  | 1 |
|  |  | Slightly worried | 1.05 ( 0.61 - 1.84 ) |  | 1.00 ( 0.54 - 1.86 ) |  | 0.92 ( 0.41 - 2.06 ) |
|  |  | Moderately worried | 1.19 ( 0.67 - 2.10 ) |  | 1.44 ( 0.76 - 2.72 ) |  | 1.03 ( 0.46 - 2.30 ) |
|  |  | Very worried | 1.88 ( 0.84 - 4.21 ) |  | 1.99 ( 0.80 - 4.94 ) |  | 2.58 ( 0.88 - 7.54 ) |
|  |  | Extremely worried | 1.02 ( 0.37 - 2.83 ) |  | 1.14 ( 0.35 - 3.77 ) |  | 1.34 ( 0.36 - 5.08 ) |
| **Mental health status during the pandemic** | | | 1.05 ( 0.97 - 1.13 ) |  | 0.98 ( 0.87 - 1.10 ) |  | 0.93 ( 0.82 - 1.06 ) |
| **Life satisfaction** | | | 1.11 ( 1.02 - 1.20 ) * |  | 1.11 ( 0.99 - 1.23 ) |  | 1.12 ( 0.99 - 1.26 ) |
| **Resilience** | | | 1.45 ( 1.11 - 1.91 ) ** |  | 1.52 ( 1.01 - 2.28 ) * |  | 1.61 ( 0.97 - 2.69 ) |
| **Willingness to receive COVID-19 vaccine** | | |  |  |  |  |  |
|  |  | Very unwilling | 1 |  |  |  | 1 |
|  |  | Somewhat unwilling | 6.99 ( 2.53 - 19.24 ) *** |  |  |  | 7.77 ( 2.66 - 22.69 ) *** |
|  |  | Neutral | 28.16 ( 10.94 - 72.53 ) *** |  |  |  | 40.73 ( 14.38 - 115.35 ) *** |
|  |  | Somewhat willing | 187.73 ( 42.44 - 830.48 ) *** |  |  |  | 304.30 ( 55.92 - 1,654.08 ) *** |
|  |  | Very willing | 70.39 ( 6.59 - 752.95 ) *** |  |  |  | 121.51 ( 6.30 - 2,344.90 ) ** |

* *p* < 0.05; ** *p* < 0.01; *** *p* < 0.001
